# Trends of preeclampsia/eclampsia and maternal and neonatal outcomes among women delivering in addis ababa selected government hospitals, Ethiopia: a retrospective cross-sectional study

**DOI:** 10.11604/pamj.supp.2016.25.2.9716

**Published:** 2016-11-26

**Authors:** Maereg Wagnew, Muluken Dessalegn, Alemayehu Worku, Josephat Nyagero

**Affiliations:** 1 Communicable and Non communicable Tropical Disease Control Department, Federal Ministry of Health in Ethiopia; 2 Amref Health Africa in Ethiopia, Monitoring Evaluation and Research Department, Addis Ababa, Ethiopia; 3 College of Health Science, School of Public Health, Department of Epidemiology and Biostatistics, Addis Ababa University, Etiopia; 4 Amref Health Africa, HQ Research Head, Kenya

**Keywords:** Preeclampsia-eclampsia, maternal-neonatal complications, HELLP, still birth, hospital

## Abstract

**Introduction:**

The burden of preeclampsia has been a major concern worldwide, particularly in developing countries such as Ethiopia. Preeclampsia is associated with substantial maternal complications, both acute and long-term. The aim of this research was to determine the magnitude and trends of preeclampsia/ eclampsia, maternal complications, and neonatal complications among women delivering babies at selected government hospitals in Ethiopia.

**Methods:**

Data were collected retrospectively by reviewing the five-year medical records for 2009 to 2013, using data abstraction tools, to identify mothers with preeclampsia/eclampsia. A total of 1,809 cases were reviewed for general characteristics of the mother, delivery details, and any complications. Descriptive analyses were employed. In addition, extended Mantel Haenszel chi square for linear trend was used to check for significance of the trends.

**Results:**

The five year average proportion of preeclampsia/eclampsia was 4.2% (95%CI 4.02%, 4.4%). The proportion of women with preeclampsia was 2.2% in 2009 and increased to 5.58% in 2013 (p<0.001), which was a 154% increase. Of the 1,809 mothers with preeclampsia/eclampsia, 36% (95%CI 33.85%, 38.28%) experienced at least one maternal complication; there was an increase of 26.5% (p<0.01) over the five year period. The main complications were HELLP (variant of preeclampsia with hemolysis, elevated liver enzymes, and low platelet count) syndrome, 257 (39.5%); aspiration pneumonia, 114 (17.5%); pulmonary edema, 114 (17.5%); and abruption placentae, 100 (15.3%). At least one neonatal complication occurred in 66.4% (95%CI 64.24%, 68.59%) of deliveries during the five-year study. A decreasing trend in neonatal complications was observed from 2009 (76%) to 2013 (66%), which showed a percentage change over time of negative 13.2%. The most common neonatal complications were stillbirths, which accounted for 363 (30.2%); prematurity, with 395 (32.8%); respiratory distress syndrome, with 456 (37.9%); and low birth weight, with 363 (30.2%).

**Conclusion:**

There was an increasing trend of preeclampsia/eclampsia and maternal complications over a five year period in selected maternity governmental hospitals. In contrast, neonatal complications experienced a significant decrease over the five-year period. It is essential to raise awareness among mothers in the community regarding early signs and symptoms of preeclampsia/eclampsia and to design a better tracking system for antenatal care programs.

## Introduction

Globally, an estimated 292,982 maternal deaths occurred in 2013. Among this global burden, 85% (245,000) occurred in sub-Saharan Africa (56%) and southern Asia (29%). More than 70% of maternal deaths are due to five major complications: hemorrhage, infection, unsafe abortion, obstructed labor, and hypertensive disorders of pregnancy, including preeclampsia and eclampsia [1]. The majority of maternal deaths (61%) occur in the postpartum period, and more than half of these take place within one day of delivery.

Preeclampsia refers to a syndrome characterized by the new onset of hypertension and proteinuria after 20 weeks gestation in a previously normotensive woman. Eclampsia refers to the development of grand mal seizures in a woman with gestational hypertension or preeclampsia [2]. The global incidence of preeclampsia (the precursor to eclampsia) ranges between 2% and 10% of pregnancies and varies greatly from one country to another. Preeclampsia is one of the leading causes of maternal mortality in Ethiopia, which is also the case in most other developing countries [3]. The World Health Organization (WHO) estimates the incidence of preeclampsia to be seven times higher in developing countries (2.8% of live births) than in developed countries (0.4%). Eclampsia increases the risk of maternal death in both developed countries (0.5 to 1.8%) and in developing countries (as high as 15%) [4]. A few studies conducted in Ethiopia showed that the prevalence of preeclampsia was around 5%; another study indicated that 0.7% of pregnancies were complicated by eclampsia [5–7].

So far, there has been no evidence of other studies that clearly indicate the trend of maternal and child health outcomes among women suffering from preeclampsia/eclampsia, both in the studied areas of Addis Ababa hospitals and in Ethiopia in general. Therefore, this study attempts to provide comprehensive information and a representative picture of potential maternal and infant health outcomes among Ethiopian women with preeclampsia/eclampsia, using different indicators, and using data extracted from hospital registrations at governmental hospitals in Addis Ababa over a five-year period.

## Methods

### Study area, design and population

Addis Ababa is the capital city of the Federal Democratic Republic of Ethiopia. According to the 2007 national census projection, the total population of Addis Ababa in July 2014 was 3.2 million, of which 47.4% were males and 52.6% were females. The total fertility rate of the city is below replacement level (1.5) and the crude death rate is 9/1000. Infant mortality rate is 40/1000 live births and the maternal mortality rate is 1/1000 live births [8].

In Addis Ababa, there are a total of 43 hospitals (21% of the total number of hospitals in the country) composed of 12 public hospitals and 31 private hospitals. In addition, there are 41 health centers (24 governmental and 7 private) and 551 private clinics. Nearly 43% of all medical doctors in the country are serving in these health facilities in Addis Ababa. The hospitals provide health care services for Addis Ababa residents and also serve as referral facilities for the nation [9]. This was a five-year, retrospective, hospital-based study conducted at selected governmental hospitals in Addis Ababa using data January 1, 2009 to December 30, 2013. All women who gave birth at selected governmental hospitals during the study period were the study population. All women who gave birth and had complete records were included; women who were diagnosed with chronic hypertension before their pregnancy were excluded from the study. The purpose of the study is to determine the magnitude and trends of preeclampsia/ eclampsia, maternal complications, and neonatal complications among women delivering babies at government hospitals.

### Sampling and study variables

Three government hospitals that offer maternal and delivery services were selected purposively based on the delivery load and the presence of fully registered data. The selected hospitals were Tikur Anbesa Specialized hospital, Zewuditu memorial hospital, and St Paul´s Hospital. All cases of preeclampsia and eclampsia found in the study hospitals were included during the data collection.

Demographic, clinical, laboratory, and management information was collected from the maternity admission registers, delivery books and operation registers on all mothers with pre-eclampsia/eclampsia who delivered babies at the selected hospitals during the study period. Maternal and neonatal complications were also collected. Information on the following was collected: Maternal variables: age, parity, gestational age, antenatal care (ANC) attendance, interventions during antenatal and perinatal period, and mode of delivery. Neonatal variables: birth weight, admission to neonatal intensive care unit, and status of the baby. Laboratory and management: data on urine protein, diagnosis on admission, and commencement of anti-hypertension or anti-convulsion drugs.

### Operational definitions of outcome variables

Maternal complications: refers to mothers who had at least one of the following complications: eclampsia, acute renal failure, stroke, intracranial hemorrhage, disseminated intravascular coagulation, HELLP syndrome, cardiac failure, abruptio placenta, aspiration pneumonia, or pulmonary edema. Neonatal complications: refers to neonates who had at least one of the following complications: preterm delivery, small-for-gestational-age, respiratory distress syndrome, low birth weight, large-for-gestational-age, macrosomia (delivery of an infant weighing >4000 grams), admission to the neonatal intensive care unit, or prenatal death.

### Measurement tools, data collection, and data analysis

A structured data-extracting (checklist) tool or form was developed and pretested before the actual data collection. Data related to maternal age, ANC follow up, severity of proteinuria, and the frequency of antenatal and intrapartum complications gravidity/parity, gestational age at presentation and delivery, intervention, mode of delivery, and health outcomes of both maternal and neonatal care were extracted. Data was collected by a nurse who had prior relevant experience. To capture cases of preeclampsia/eclampsia in all selected hospitals, medical records and delivery registry books for the study period were reviewed.

The extracted data was cleaned, checked for accuracy, consistency, entered using Epi Info version 3.5.1 software and analyzed using SPSS version 22 and Open Epi version 2.2. Both descriptive and analytical statistical procedures were employed. The analysis presented in the epidemiological trends followed the following steps: proportions were computed by dividing those preeclampsia/eclampsia values with the respective total delivery/denominator for each specific year; proportions were computed for maternal and neonatal outcomes among preeclampsia/eclampsia cases for each year; trends of maternal and neonatal outcomes among preeclampsia/eclampsia cases for each year were analyzed; when appropriate, numeric summaries were used to describe the nature of the trend in the specific trend variable (X2), stratification was done by year (Mantel Haenszel chi square was used) to assess significant linear trend over time.

### Ethical considerations

Ethical clearance was obtained from the School of Public Health, College of Health Science, Addis Ababa University. Official letter of cooperation was taken from the School of Public Health to the respective departments and medical directors of the hospitals where the study was undertaken and another ethical clearance was obtained from the IRB of each three hospitals. Consent was obtained from each hospital to retrieve the five-year data. Anonymity and confidentiality were ensured for information obtained from the charts.

## Results

### Characteristics of the retrospective data

As presented in Table 1, the analysis included a total of 42,963 deliveries in five years from 2009 to 2013. Of those, 79% of cases were from the central city of Addis Ababa by residence and the rest came from different parts of the region, mainly from Oromia region.

**Table 1:** Characteristics of mothers with preeclampsia/eclampsia from 2009-2013 in government hospitals, Addis Ababa, Ethiopia (N=1809)

Variable	Response	Frequency (%)	Mean (±SD)
Year of the data	2009	160 (8.8)	
	2010	317 (17.5)	
	2011	359 (19.8)	
	2012	446 (24.7)	
	2013	527 (29.1)	
Maternal age (years)	<20	104 (5.7)	26.75 (±5.08)
	20-24	499 (27.5)	
	25-29	663 (36.6)	
	30-34	356 (19.7)	
	35^+^	187 (10.5)	
Parity	Primipara	959 (53.0)	
	Multipara	850 (47)	
Medical related history	Yes	79 (4.4%)	
	No	1730 (95.6)	
Past pregnancy history	Yes	492 (27.2)	
	No	1317 (72.8)	
Gestational age (weeks)	20-28	112 (6.2)	
	29-36	737 (40.7)	
	37-41	897 (49.6)	
	>42	63 (3.5)	

In the five-year study, among total deliveries, 1,809 cases of preeclampsia/eclampsia were registered, with a five-year average proportion of 4.2% (95%CI: 4.02%, 4.4%). Of these, 1,424 (78.1%) were severe preeclampsia, 313 (17.3%) were eclampsia, and 84 (4.6%) were mild preeclampsia.

The mean maternal age of women with preeclampsia/eclampsia was 26.75 (±5.08) years, with 663 (36.6%) women in the age range from 25 to 29 years old. With regard to parity, 959 (53%) were nulliparous and 39 (2.2%) were parity five and above. The mean gestational age at presentation was 35.83 (±3.7) weeks.

Based on the five-year data of mothers who had preeclampsia/eclampsia, 79 (4.4%) had chronic medical history, mainly HIV and AIDS with 43 cases (54.4%) and diabetes with 16 cases (20.3%). A history of at least one complication during a past pregnancy was reported for 492 (27.2%) women. The major complications were abortion, 285 cases (57.9%); severe preeclampsia, 140 cases (28.5%); Cesarean section(C/S) delivery, 96 cases (19.5%); still birth, 60 cases (12.2%), neonatal death, 24 cases (4.9%); and eclampsia, 14 (2.8%) cases.

### Condition of the mother at the time of admission

A family history of hypertension was reported for 101 (5.6%) women and a family history of preeclampsia was reported for only 0.7%. The mothers’ mean systolic blood pressure was 157±16.1 mmHg, ranging from 120 to 230 mmHg, and the mean diastolic blood pressure was 103±10.85 mmHg, ranging from 80 to 160 mmHg. The majority of mothers had edema (1589 women (87.8%)) and proteinuria, (1,725 women (95.4%)).

Among mothers who had preeclampsia and eclampsia present at admission, many complained of one or more of the following signs and symptoms: 88.8% complained of headache, 49.5% of blurred vision, 35.9% of epigastric pain, and 17% of abnormal body movement.

### Characteristics of the mothers during the time of delivery

As presented in Table 2, there were 387 (21.4%) mothers who did not have antenatal follow up. Based on the diagnosis of the mothers, the majority of cases, 1,412 (78.1%), were severe preeclampsia. With regard to labor, 423 (23.4%) were induced/terminated and the main indication, with 279 cases (66%), was severe preeclampsia/term pregnancy.

**Table 2:** Characteristics of mothers with preeclampsia/ eclampsia during delivery in 2009-2013 government hospitals, Addis Ababa

Variable (N=1809)	Response	Frequency n (%)
Antenatal care follow up	Yes	1422 (78.6%)
	No	387 (21.4%)
Diagnosis	Mild preeclampsia	84 (4.6%)
	Severe preeclampsia	1412 (78.1%)
	Eclampsia	313 (17.3%)
Presentation	Cephalic	1661 (91.8%)
	Breech	134 (7.4%)
	Transverse	14 (0.8%)
Labor induced or terminated	Yes	423 (23.4%)
	No	1386 (76.6%)
Indication for induction/termination	Severe preeclampsia and gestational age >34	7 (1.7%)
	Severe preeclampsia pregnancy	279 (66.0%)
	Abruption placenta	11 (2.6%)
	HELLP syndrome	10 (2.4%)
	Fetal distress	79 (18.7%)
	Eclampsia	18 (4.3%)
Mode of delivery	Spontaneous vaginal delivery	905 (50.0%)
	Cesarean section	800 (44.3%)
	Instrumental delivery	84 (5.6%)

The data on the mode of delivery showed that half of the deliveries, 905 (50%), took place by spontaneous vaginal delivery and 800 (44.3%) were C/S deliveries. The rest of the deliveries were associated with instrumental (forceps + vacuum) and breech extraction. Fetal distress and failed induction were the most common indications for C/S delivery.

### Management of the preeclampsia/eclampsia mother

Among the 1,809 women with preeclampsia/eclampsia, 1,340 (74.1%) of them received MgSo4 as a means for managing the illness and the majority (96.3%) of the mothers’ blood pressure (BP) lowered to less than 160/100 mmHg at discharge. In addition to these, many mothers were kept in the hospital and received anti-hypertensive treatment.

### The five-year (2009-2013) pattern of maternal and neonatal complications

Among the 1,809 women with preeclampsia/eclampsia, 652 (36%) had maternal complications. HELLP syndrome was the most frequent complication observed in mothers, with 257 (39.5%) cases. Aspiration pneumonia 114 (17.5%), pulmonary edema 114 (17.5%), and abraptio placenta 100 (15.3%) were also frequent maternal complications (Table 3).

**Table 3:** Pattern of maternal and neonatal complications among preeclampsia/ eclampsia in 2009-2013 in government hospitals in Addis Ababa, Ethiopia

Variable	Response	Frequency (%)
Maternal complication (N=1809)	Yes	652 (36.0%)
	No	1157 (64.0%)
Maternal complications (N=652)	HELLP syndrome	257 (39.5%)
	Aspiration pneumonia	114 (17.5%)
	Pulmonary edema	114 (17.5%)
	Abruptio placentae	100 (15.3%)
	Post-partum hemorrhage	56 (8.6%)
	Institutional maternal death	6 (0.9%)
	Other	5 (0.8%)
Birth weight (N=1809)	<1500 grams	301 (16.6%)
	1500-2500 grams	743 (41.1%)
	>2500 grams	765 (42.3%)
Neonatal complication (N=1809)	Yes	1202 (66.4%)
	No	607 (33.6%)
Most common neonatal complications+(N=1202)	Low birth weight	532 (44.2%)
	Respiratory distress syndrome	456 (37.9%)
	Premature	395 (32.8%)
	Still birth	363 (30.2%)
	Asphyxia	102 (8.5%)
Outcome of early neonatal (N=1446)	Alive	1296 (91.7%)
	Dead	150 (8.3%)

+ More than one complication may have been reported

Amongst all deliveries, 1,202 (66.4%) (95%CI 64.24% - 68.59%) had at least one neonatal complication. The rate of birth weight <1500 grams was 301 (16.6%) and 1500-2500 grams was 743 (41.1%). There were 363 (30.2%) still births. Among still birth cases, 243 (66.9%) and 115 (31.7%) were among mothers who had severe preeclampsia and eclampsia, respectively. The most common causes of neonatal complications were prematurity, with 395 cases (32.8%); respiratory distress syndrome, 456 cases (37.9%); and low birth weight, 363 cases (30.2%). There were also 756 (41.8%) neonatal intensive care unit admissions and, from the total number of deliveries, there were 150 (8.29%) early neonatal deaths (Table 3).

### Trends of preeclampsia/eclampsia among the total deliveries

**Figure 1:**
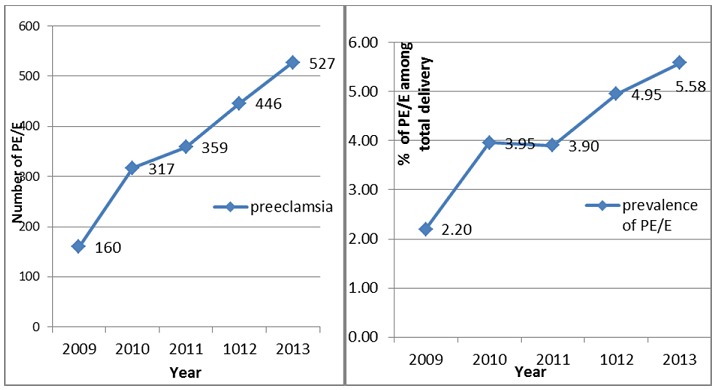
A) the Number of preeclampsia/eclampsia 2009 to 2013; B) the proportion of PE/E from 2009 to 2013 (X^2^_MH_=121, P<0.001)

As shown in Figure 1, the incidence of preeclampsia/eclampsia increased yearly from 2009 to 2013. The proportion of preeclampsia was 2.2% in 2009 and increased to a proportion of 5.58% in 2013 (p<0.001). The percentage increase over five years was 154%. The test of Mantel-Haenszel odds ratios, the rate of preeclampsia/eclampsia as years passed from 2009 to 2013 increased by 2.63 times and the test showed a significant change in linearity (X^2^_MH_=121, p<0.001). According the five year data review, severe preeclampsia dominated in all five years and had greater changes throughout the five-year period, while mild preeclampsia had the lowest incidence in the five-year study, and the change was minimal.

### Trends of maternal complications

**Figure 2:**
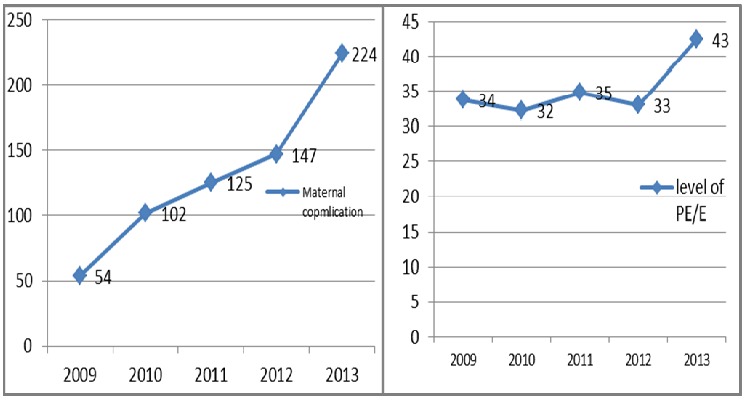
A) the number of maternal complications from 2009 to 2013; B) the proportion of maternal complications among PE/E from 2009 to 2013

Among mothers who had preeclampsia/eclampsia, 652 (36.04%) (95%CI: 33.85% - 38.28%) had at least one maternal complication. The number of women with at least one type of maternal complication increased from 2009 (54) to 2013 (224) (Figure 2). Although this increase in number of complications may be related to an increase in the number of preeclampsia/eclampsia cases; however, the proportion of maternal complications was lowest in 2010 and 2012, but increased from 33% in 2012 to 43% in 2013. The percentage change in maternal complications over time was 26.5%. The trend of these graphs has a global significance of linearity (X^2^_MH_=7.55, p<0.006). The maternal complication rate in 2013 was 1.45 times the rate in 2009.

### Trends of neonatal complications

**Figure 3:**
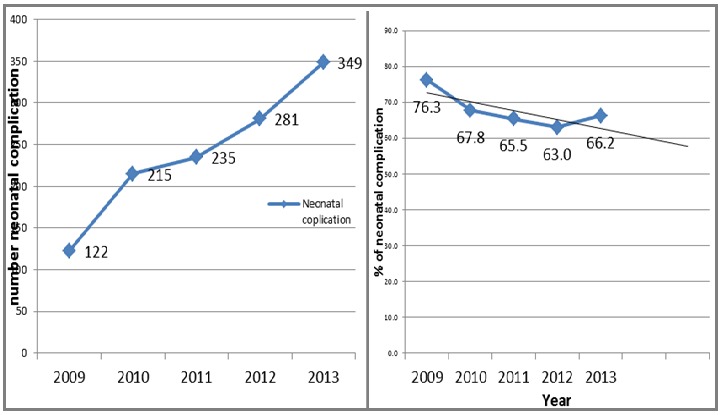
Trends of neonatal complications in 2009-2013 in three government Hospitals, Addis Ababa

As shown in Figure 3, the number of neonatal complication seems to have increased. However, when the analysis took into account cases of preeclampsia/eclampsia as the denominator (proportion), the evidence showed that the neonatal complication rate decreased from 76% in 2009 to 66% in 2013 (change of negative 13.2%). This reduction in neonatal complications was significant and has a decreasing linearity (p<0.04028, X^2^_MH_=4.21).

## Discussion

Preeclampsia contributes to maternal and perinatal morbidity and mortality and remains a major obstetric concern and a significant global public health threat, especially in developing countries like Ethiopia [10, 11]. Studies done in Ethiopia and elsewhere indicate that the prevalence of maternal and neonatal morbidity and mortality associated with preeclampsia/eclampsia has increased from year to year, especially in developing countries [12, 13]. In this study, the magnitude of preeclampsia/eclampsia was 4.2%, which is consistent with a prior study done in Ethiopia at Tikur Anbesa Hospital (magnitude of 5%) [14] though the denominator was has huge difference (42,963 Vs. 1941). The trend of preeclampsia/eclampsia shows a statistically significant (p<0.01) percentage increase of 154% from 2009 to 2013, which is consistent with another study conducted in Ethiopia [12, 13]. This increase might be related to an improvement in case detection rates due to special emphasis in the country or for selected hospital compared with previous years, or might be the result of an actual increase in the number of preeclampsia/eclampsia cases, which would require further large scale studies. As many other studies, including some performed in Ethiopia [15], have shown, severe preeclampsia, with a rate of 78%, was more common than mild preeclampsia and eclampsia over the five-year study.

In this study, maternal complication was present in 36% of the preeclampsia/eclampsia cases. This is consistent with a study conducted among a similar study group in Nigeria, with a presence of maternal complication for 39% of the cases [16]. Furthermore, we noted a decrease in the proportion of maternal complications within cases of preeclampsia/eclampsia in the years 2010 and 2011. This decrease might be attributable to the introduction of magnesium-sulphate (MgSo4) to treat preeclampsia/eclampsia in those hospitals, leading to a reduction in the prevalence of maternal complications [3]. However, apart from the years 2010 and 2011, the percentage increase of maternal complications over time was 26.5%. According to the study on maternal complication [17], with a focus on maternal mortality done in Ethiopia, the proportion of complications due to preeclampsia/eclampsia increased, which is consistent with results obtained in our study. This might be related to the three delays (delay in the decision to seek care, delay in reaching the health facility, and delay in health service provision), which can result in a higher incidence of complications. For example, among women who have preeclampsia/eclampsia more than 64% of mothers have late booking experience. However, this is difficult to explain and requires further verification from intervention programs.

Women with preeclampsia and eclampsia showed a 3- to 25-fold higher risk of severe complications such as abruptio placentae, HELLP syndrome, disseminated intravascular coagulation, pulmonary edema, and aspiration pneumonia. This study also consistently identified those as the most common complications among mothers with preeclampsia/eclampsia [18]. Preeclampsia is generally known to affect primigravida women, which is consistent with our findings showing that more than half of the cases were amongst primigravida women (53%). The mean gestational age at delivery was 35.83 (±3.7) weeks and this was consistent with results from the a study conducted in the Eastern Cape (35.16 ±3.80 weeks) [19]. This might increase the percentage of premature neonates and percentage of respiratory distress syndromes, leading preterm delivery to become more common.

The neonatal complication rate has shown a statistically significant decrease from 2009 to 2013, dropping from 76% to 66% during this time. The change in neonatal complication rates over time was of negative 13.2%. This result may be attributed to better attention given to newborn care, in order to decrease neonatal mortality and morbidity to achieve the sustainable developmental goals on a national level and worldwide. Methods for better newborn care are, for example, the establishment and strengthening of the neonatal intensive care unit and the introduction of the MgSo4 drug to reduce and control maternal and neonatal morbidity and mortality, as well as other possible factors that may have contributed to this reduction in neonatal complications.

In this study, 66.4% of neonates had at least one complication; the majority of complications were low birth weight, respiratory distress syndrome, premature birth, and still birth. The rate of neonatal complications is notably higher in women with preeclampsia/eclampsia.

Overall, the findings of this study have important policy implications for health interventions on preeclampsia/eclampsia, maternal complications, and neonatal complications based on those selected hospitals and many other hospitals that have similar nature. This has an important public health significance regarding maternal and neonatal health; therefore, special attention should be given to mothers and neonates to halt the progression of preeclampsia/eclampsia and its consequences. In the long run, these problems may be alleviated by integrating efforts from different sectors. However, short term efforts must be implemented in the interim period including; raising awareness at community level among mothers during ANC visits regarding early signs and inform them of the referral system to help reduce the “three delays.” It is essential at facility level to design a better tracking system for ANC and providing health facilities with MgSo4 which could prevent or reduce convulsions and severe outcomes in mothers and neonates. Strengthening and establishing neonatal intensive care unit in health facilities could be an important factor in reducing the severity of neonatal complications. Additionally, the policy makers and implementers should review the recommendations for management of preeclampsia/ eclampsia cases and adjust policies to the present needs. Finally, further community-based studies are encouraged in order to identify the causes behind this increase in preeclampsia/eclampsia cases and maternal complications.

Our study had the following limitations: The trend was limited to five years due to unavailability of registration in hospitals and retrospective nature of the study by itself. The study was also limited to hospitals and unable to assess the community-based burden, which may be higher.

## Conclusion

Even though varies interventions were introduced at a country level in the past five years, the proportion of preeclampsia/eclampsia cases in some governmental hospitals significantly increased over time from 2009 to 2013. The rate of maternal complications during birth has remained relatively stable since the introduction of MgSO4 for preeclampsia/eclampsia management; however, the rate of complications among preeclampsia/eclampsia cases has experienced significant increase overtime mainly in the later years. Neonatal complications arising from deliveries by pre-eclamptic/ eclamptic mothers have significantly declined overtime. In conclusion, based on our research findings and using preeclampsia/ eclampsia as an indicator of maternal health, the current state of maternal health some governmental hospitals in and hospitals with similar nature in Ethiopia was far from achieving the millennium developmental goals and in need of big assignment for sustainable development goals. In addition, though early neonatal complications have decreased over time, the proportion remains very high. Attention should be focused on raising awareness at community level and strengthening health facilities for early detection/ prevention and management of pre-eclampsia/ eclampsia by equipping health facilities to manage the complication related to the complication of mothers and children.

### What is known about this topic

- Neonatal mortality has been decreasing similarly neonatal complications arising from deliveries by pre-eclamptic/ eclamptic mothers have significantly declined overtime;
- Maternal mortality in Ethiopia showed a decrement at country level;
- In Ethiopia, a delay in health seeking is the common challenges in maternal, neonatal health.

### What this study adds

- The rate of preeclampsia/eclampsia cases in Tikur Anbesa, Saint Paul and Ghandi hospitals increased year after year where we didn’t have any evidence in the country in the past in study areas and other;
- Though in Ethiopia in general and selected governmental hospitals in particular, there are many interventions introduced the rate of maternal complications has remained relatively stable since the introduction of MgSO4 for preeclampsia/ eclampsia management;
- In this study, in maternal and neonatal health outcomes among women who have preeclampsia/ eclampsia is limited to the community rather the service delivery and tracking mechanisms at health facility and lack of clear guidelines in providing specific services has huge role.

## References

[1] WHO (2012). Trend In Maternal Mortality: 1990 to 2010: WHO, UNICEF, UNFPA, and The World Bank estimates..

[2] Sibai BM (2005). Diagnosis, prevention, and management of eclampsia. Obstet Gynecol..

[3] Federal Ministry of Health (2010). National Baseline Assessment for Emergency Obstetric and Newborn Care Final Report 2008..

[4] WHO (2009). Monitoring Emergency Obstet ric Care: a Handbook..

[5] Teklu S, Gaym A (2006). Prevalence and clinical correlates of the hypertensive disorders of pregnancy at Tikur Anbessa Hospital Addis Ababa, Ethiopia. Ethiop Med J..

[6] Mekbeb T, Ketsela K (1991). Pre-eclampsia/ eclampsia at Yekatit 12 Hospital, Addis Ababa, Ethiopia (1987-1989). East Afr Med J..

[7] Abate M, Lakew Z (2006). Eclampsia a 5 years retrospective review of 216 cases managed in two teaching hospitals in Addis Ababa. Ethiop Med J..

[8] Population Census Commission (2007). The Population and Housing Census of Ethiopia. Statistical Report for Ethiopia.

[9] Misganaw A (2012). Ethiopian Ministry of Health: Health and Health Related Indicators: validity of Verbal Autopsy Method to determine causes of death among adults in the urban setting of Ethiopia. BMC Med Res Methodol..

[10] WHO (2002). Global Program to Conquer Preecclampsia/Ecclampsia..

[11] McClure EM (2009). Stillbirth in developing countries: a review of causes, risk factors and prevention strategies. Journal of Maternal-Fetal and Neonatal Medicine..

[12] Asheber Gaym (2011). Ethiopian National EmONC Assessment Team, Disease burden due to pre-eclampsia/eclampsia and the Ethiopian health system's response, 2011. International Federation of Gynecology and Obstetrics..

[13] Khalid Khan S (2006). WHO analysis of causes of maternal death: a systematic Review. Lancet..

[14] Eyob Berihun, Asheber Gaym (2007). Risk factors for mortality among eclamptics admitted to the surgical intensive care unit at Tikur Anbessa Hospital, Addis Ababa, Ethiopia. Ethiopian Journal of Reproductive Health..

[15] Zenebe Wolde, Hailemariam Segni, Mirkuzie Woldie (2011). Hypertensive disorders of pregnancy in jimma University specialized hospital. Ethiop J Health Sci November..

[16] Tukur Jido A (2012). Ecalmpsia: maternal and fetal outcome. African Health Sciences..

[17] Ahmed Abdella (2010). Maternal Mortality Trend in Ethiopia. Ethiop J Health Dev..

[18] Zhang J, Meikle S, Trumble A (2003). Severe maternal morbidity associated with hypertensive disorders in pregnancy in the United States. Hyper-tens Pregnancy..

[19] Olumide Ojodun The prevalence of hypertensive complications of pregnancy in Dora Nginza Hospital, Port Elizabeth, Eastern Cape..

